# Post-genomics revolution in the design of premium quality rice in a high-yielding background to meet consumer demands in the 21st century

**DOI:** 10.1016/j.xplc.2021.100271

**Published:** 2021-12-28

**Authors:** Nese Sreenivasulu, Changquan Zhang, Rhowell N. Tiozon, Qiaoquan Liu

**Affiliations:** 1 Consumer Driven Grain Quality and Nutrition Unit, Rice Breeding and Innovation Platform, International Rice Research Institute, Los Baños 4030, Philippines; 2 Key Laboratory of Plant Functional Genomics of the Ministry of Education, Jiangsu Key Laboratory of Crop Genomics and Molecular Breeding, College of Agriculture, Yangzhou University, Yangzhou 225009, China; 3 Jiangsu Co-Innovation Center for Modern Production Technology of Grain Crops, Jiangsu Key Laboratory of Crop Genetics and Physiology, Yangzhou University, Yangzhou 225009, China; 4 Max Planck Institute of Molecular Plant Physiology, Am Mühlenberg 1, 14476 Potsdam-Golm, Germany

**Keywords:** amylose, amylopectin, eating and cooking quality, genetics, genome editing, texture

## Abstract

The eating and cooking quality (ECQ) of rice is critical for determining its economic value in the marketplace and promoting consumer acceptance. It has therefore been of paramount importance in rice breeding programs. Here, we highlight advances in genetic studies of ECQ and discuss prospects for further enhancement of ECQ in rice. Innovations in gene- and genome-editing techniques have enabled improvements in rice ECQ. Significant genes and quantitative trait loci (QTLs) have been shown to regulate starch composition, thereby affecting amylose content and thermal and pasting properties. A limited number of genes/QTLs have been identified for other ECQ properties such as protein content and aroma. Marker-assisted breeding has identified rare alleles in diverse genetic resources that are associated with superior ECQ properties. The post-genomics-driven information summarized in this review is relevant for augmenting current breeding strategies to meet consumer preferences and growing population demands.

## Teaser

Conventional breeding strategies have led to the development of improved elite rice varieties, albeit with limitations in grain quality traits. Therefore, modern molecular breeding strategies have been used to identify new genes related to eating and cooking quality (ECQ). This review summarizes different post-genomic tools that can augment current breeding strategies to meet consumer preferences and growing population demands.

## Introduction

**Figure 1:**
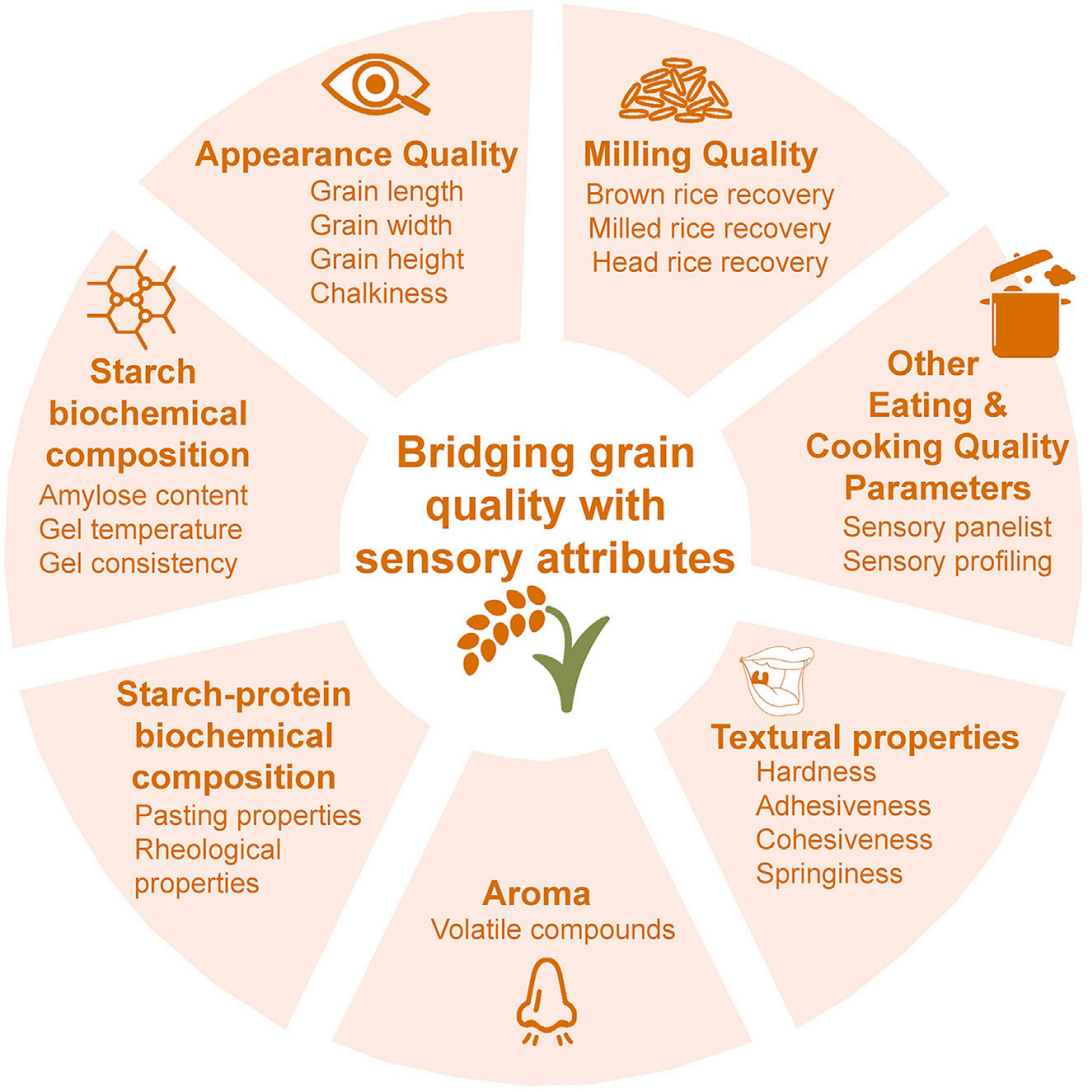
Holistic approaches to eating and cooking quality.

Rice grain quality is a complex trait that reflects aroma, taste, milling, appearance, and eating and cooking quality (ECQ) (Anacleto et al., 2015; Custodio et al., 2019). In recent years, ECQ, also known as sensory quality, has become one of the most important considerations for rice producers aiming to capture consumer preferences in the marketplace. To add premium value, both ECQ (e.g., textural attributes such as stickiness, elasticity, and hardness) and aroma must be addressed (Li and Gilbert, 2018). To achieve premium quality, holistic approaches are needed to link biochemical variation in starch properties, proteins, and lipids to textural and sensory properties (Figure 1). It is difficult to evaluate rice grain quality preferences from a global perspective because rice quality differs between countries and target zones (Custodio et al., 2019). For instance, consumers in Southern China, India, Bangladesh, Sri Lanka, and Pakistan prefer long, slender grains with a range of fluffy to harder textures. To meet these requirements, breeders produce varieties with intermediate to high amylose content. Consumers in northern China, Japan, and South Korea prefer medium grains with a relatively soft texture, and varieties with a low amylose content have therefore been developed.

Rice grains are composed of approximately 80%–85% starch, 4%–10% protein, and 1% lipid, and the range of genetic variability in biochemical composition plays a role in determining rice ECQ. Starch is the primary component that plays a vital role in determining rice ECQ properties (Tian et al., 2009). Rice starch is generally composed of two components, amylose and amylopectin; its composition varies among varieties, contributing to variability in pasting viscosity and texture (Buenafe et al., 2021). Consistent data have shown that amylose content (AC) within the rice endosperm is the most critical factor that determines the physicochemical properties of starch and its end-use quality (Li and Gilbert, 2018). In particular, among intermediate- to high-amylose varieties, it is important to identify those with grains that do not undergo retrogradation (i.e., the rice remains soft after cooking and cooling). This trait is traditionally measured by gel consistency (GC) and rapid viscosity properties, such as final viscosity (FV) and set back (Buenafe et al., 2021). In addition, gelatinization temperature (GT) is another crucial factor that affects rice ECQ. Rice grain with high GT is thought to require longer cooking times, and the texture of the cooked rice tends to be less sticky, especially when it is cooled (Zhang et al., 2018a; Nakata et al., 2018).

Protein is the second principal component of rice grain, and it is the key factor for the evaluation of rice nutritional quality (Zhao et al., 2020a). Grain protein content (PC) varies among rice varieties, ranging from 4.9% to 19.3% in indica rice and from 5.9% to 16.5% in japonica rice (Yang et al., 2015). In recent years, progress has been made in revealing the relationships between PC (specific proteins) and rice ECQ. Prior research indicates that higher PC generally leads to reduced ECQ, perhaps owing to the high positive correlation between PC and deteriorated texture in cooked rice (Li et al., 2019). The structure of starch is thought to determine the overall hardness of cooked rice, whereas PC and composition determine the surface hardness. For example, rice grain with a low PC generally shows better ECQ (Li et al., 2019; Zhang et al., 2021a). Thus, the genetic manipulation of PC-related genes is essential for improving the ECQ of rice.

In the rice endosperm, lipids are minor nutrients compared with starch and protein, and the preferential accumulation of lipids is known to occur in the aleurone and the embryo during desiccation (Huang et al., 2020a). The most abundant lipid groups are glycerolipids in the form of triacylglycerols, followed by glycerophospholipids and free fatty acids; these components comprise 84.7%–86.0%, 6.5%–6.7%, and 4.2%–4.6% of the total lipids in well-milled rice, respectively (Yoshida et al., 2011). Lipids can form complexes with amylose and amylopectin, thereby affecting the texture of cooked rice (Tong et al., 2015; Concepcion et al., 2020). Interestingly, Concepcion et al. (2018) found that the unsaturated fatty acids in milled rice contribute to its fragrance, and rice grains from high-quality fragrant varieties tend to contain a high level of unsaturated fatty acids (Concepcion et al., 2018). Thus, it is essential to understand the genetic factors that govern seed storage products (amylose–amylopectin ratio, seed storage proteins, and phospholipids) and their influence on ECQ traits in order to breed varieties that match specific market segments.

Aroma is one of the most significant quality traits of rice, and varieties with aromas found in jasmine and basmati rice types command a higher price in the marketplace. It is apparent that the volatile compound 2-acetyl-1-pyrroline (2-AP) is the main compound associated with rice grain fragrance, lending a popcorn-like aroma, but the genetic variations underlying other diverse aromas, such as cracker-like, roasted, sweet nutty, and milky aromas, are not known (Custodio et al., 2019). An overlapping metabolite pathway study identified volatile compounds such as hexanal, octanal, nonanal, (E)-2-octenal, decanal, 1-heptanol, 1-octanol, and acetoin as major aroma-active compounds in rice grains (Zhao et al., 2020b; Jie et al., 2021). However, it remains unclear how these volatiles contribute to the aroma of rice.

Acceptance of new rice genotypes demanded by the rice value chain hinges on their potential for higher yields and depends on premium value genotypes that can satisfy consumer preferences for grain and nutritional quality. Recent advances in grain quality profiling and -omics technologies have provided efficient approaches for the identification of key genes and biochemical markers for rice quality traits. In this review, emphasis is given to a holistic understanding of the molecular genetic components that influence ECQ attributes, and we discuss genomics-aided information that links starch biochemistry with sensory evaluation to assist with selection during breeding.

## Genetic basis of rice ECQ improvement

**Figure 2:**
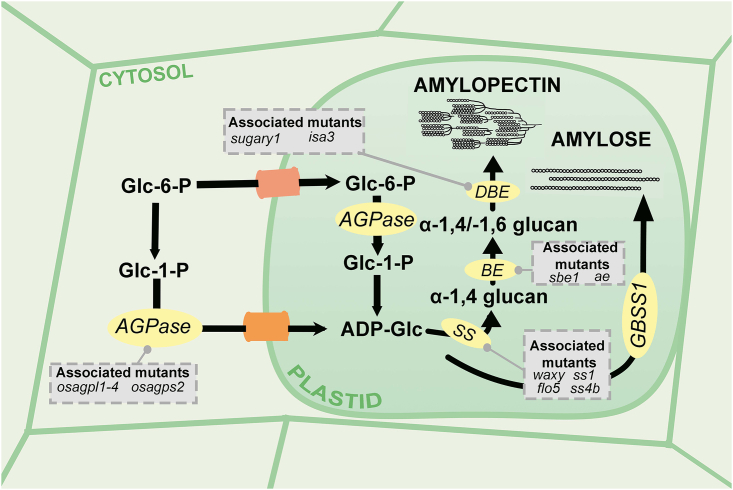
Starch biosynthesis and key enzymes with their associated mutants.

With innovations in starch genetics, it is now possible to control starch structure through modification of the amylose–amylopectin ratio. The structure of starch may be conceptually simple, but its biosynthesis is complex. Starch accumulates in the rice endosperm during the grain-filling period and is mainly produced by the concerted efforts of four enzymes: ADP-glucose pyrophosphorylase (AGPase), starch synthases (SSs), starch branching enzymes (SBEs), and debranching enzymes (DBEs). Figure 2 shows starch biosynthesis and mutants of important starch synthesis enzymes. The post-translational modification of these enzymes through phosphorylation, alternative splicing, and allosteric changes plays a vital role in modifying starch content and altering the amylose–amylopectin composition (Huang et al., 2020b; Adegoke et al., 2021). Table 1 shows various mutants with altered physicochemical properties.

**Table 1:** Rice mutants and their corresponding properties.

Mutants	Chr	AAC	Amylopectin CLD	Pasting property	Thermal property	Key information	Refs.
*ae* (*aeae/WxWx*)	2	26.5%–35% (high amylose)	DP ≦ 17 ↓, DP 18–36 ↑, DP ≧ 38 ↑	–	–	The *ae* mutation led to a dramatic reduction in the activity of BEIIb. The activity of SSI was significantly lower in the *ae* mutant than in the wild type. This implies that the mutation had a pleiotropic effect on SSI activity.	Yano et al. (1985), Nishi et al. (2001)
*dull1*	10	5% (very low amylose)	–	–	–	*Du1* encodes a novel Prp1 protein that regulates starch biosynthesis by affecting the splicing of Wxb pre-mRNAs in rice.	Zeng et al. (2007)
*dull3*	2	7% (very low amylose)	–	–	–	*Du3* encodes the rice homolog of a cap-binding protein 20 kDa subunit (CBP20) and plays a role in pre-mRNA splicing, RNA nuclear export, and nonsense-mediated decay.	Isshiki et al. (2008)
*flo2*	4	11.2%–11.9% (low amylose)	DP 9–21 ↓, DP 22–38 ↑, DP ≥ 38 ↓	HPV: 68–79 (↓) PV: 132.46–164.33 (↓) CPV: 106.67–121.71 (↓) BD: 64.46–85.25 (↓) SBV: –25.79–42.63 (↓) CSV: 38.67–43.46 (↓)	–	*Flo2* encodes a tetratricopeptide repeat domain-containing protein. It influences rice grain size and starch quality by affecting storage substance accumulation in the endosperm. It perturbed the expression of genes such as *OsAGPL2*, *OsAGPS2b*, *OsGBSSI*, *OsBEI*, *OsBEIIb*, *OsISA1*, and *OsPUL*. The mutation conferred dull rather than floury grains.	Wu et al. (2015)
*flo4*	5	15.2%–16.5% (low amylose)	no significant changes observed	–	–	*Flo4* encodes a C4-type pyruvate orthophosphate dikinase (OsPPDKB) and modulates carbon metabolism during the grain-filling period.	Kang et al. (2005)
*flo6* (new mutant of OsAPL2)	3	14.53% (low amylose)	DP 6–8 ↑, DP 9–15 ↓, DP 16–40 ↑	PV: ↓ BD: ↓ CPV: ↓	GT: 62.14 ± 0.06 (↓) *T*_o_: 50.42 ± 0.12 (=) *T*_p_: 69.84 ± 0.30 (↓) *T*_c_: 86.77 ± 0.18 (↓) Δ*H*_g_: –9.67 ± 0.15 (↑)	*Flo6* encodes a CBM48 involved in compound granule formation and starch synthesis in the rice endosperm.	Peng et al. (2014b), Zhang et al. (2021c)
*flo7*	12	15.4% ± 0.3% (low amylose)	DP6–7 ↓, DP 8–14 ↑, DP15–55 ↓	–	–	*Flo7* encodes a regulator of starch synthesis and amyloplast development that is essential for peripheral endosperm development in rice.	Zhang et al. (2016)
*flo8*	9	∼13% (low amylose)	DP7–9 ↑, DP10–13 ↓, DP14–16 ↑, DP ≥ 17 ↓	PV: ↓ BD: ↓ CPV: ↓	–	*Flo8* encodes UDP–glucose pyrophosphorylase 1, which affects the synthesis and structure of starch in the rice endosperm.	Long et al. (2017)
*isa3*	9	22.9 ± 0.1 (intermediate amylose)	DP 3–7 ↓, DP 10–19 ↓	PV: 512.5 ± 0.1 (↓) BD: 344.1 ± 2.7 (↓) PT: 66.5 ± 0.0 (=)	*T*_o_: 46.9 ± 0.3 (↓) *T*_p_: 61.1 ± 0.2 (↓) *T*_c_: 72.6 ± 0.2 (↓) Δ*H*_g_: 6.0 ± 0.1 (↑)	*Isa3* facilitates starch metabolism, which also affects plastid morphogenesis.	Yun et al. (2011)
*M14*	5	13.8% (low amylose)	DP 6–12 ↑, DP 13–50 ↓	PV: ↓ BD: ↓ CPV: ↓	*T*_o_: 57.7 ± 0.2 (↓) *T*_p_: 65.7 ± 0.3 (↓) *T*_c_: 72.1 ± 0.1 (↓) Δ*H*_g_: 14.4 ± 0.3 (↑)	The mutation of *OsPPDKB* led to significant downregulation of *GBSSI* expression.	Zhang et al. (2018b)
*osbt1*	2	12% (low amylose)	DP 7–17 ↑, DP 18–30 ↓	PV: ↓ BD: ↓ CPV: ↓ (specific values were not reported)	*T*_o_: 60.5 (↓) *T*_p_: 69 (↓) *T*_c_: 76 (↓)	*OsBT1* encodes a putative ADP-glucose transporter. It localizes in the amyloplast envelope membrane and plays a role in starch synthesis and the formation of compound starch granules.	Li et al. (2017)
*pul*	4	Value not reported, no significant impact on AAC	DP 7–12 ↑, DP ≤ 13 ↑	–	*T*_o_: 53.6 ± 1.4 (↓) *T*_p_: 61.0 ± 0.5 (↓) *T*_c_: 66.8 ± 0.3 (↓) Δ*H*_g_: 4.9 ± 0.8 (↓)	The activity of PUL was correlated with the severity of the *sug1* phenotype.	Yun et al. (2011)
*rsr1*	5	19% (low amylose)	DP 5–8 ↑, DP 9–17 ↓, DP 18–38 ↑	–	*T*_o_: 44.94 ± 0.34 (↓) *T*_p_: 57.64 ± 1.55 (↓) *T*_c_: 63.46 ± 1.31 (↓)	*RSR1* encodes an APETALA2/ethylene-responsive element binding protein family transcription factor. It negatively regulates the expression of type I starch synthesis genes, and its deficiency results in the improved expression of starch synthesis genes in seeds.	Fu and Xue (2010)
*sbe1*	6	18.9% (low amylose)	DP ≤ 10 ↑, DP 12 to 21 ↓, DP 24 to 34 ↑, DP ≥ 37 ↓	-	*T*_o_: 45.1–45.9 (↓) *T*_p_: 55.0–55.4 (↓) *T*_c_: 63.9–64.7 (↓) Δ*H*_g_: 8.3–10.0 (↓)	Its mutation did not change the amount of starch but altered the fine structure of amylopectin.	Satoh et al. (2003)
*ss1*	6	Value not reported, no significant impact on AAC	DP 6–7 ↑, DP 8–12 ↓, DP 16–19 ↑	PV: ↓ BD: ↓ CPV: ↓ (specific values were not reported)	*T*_o_: ↓ *T*_p_: ↓ *T*_c_: ↓ (specific values were not reported)	*SSI* may play a distinct role in starch biosynthesis. Its deficiency is associated with a direct change in the structure of starch granules.	Fujita et al. (2006)
*sug2*	11	21.81 ± 1.04 (intermediate amylose)	DP ≤ 8 ↑, DP 8–25 ↑, DP 25–35 ↓, DP 35–67 ↑, DP > 67 ↓	–	*T*_o_: 53.86 ± 0.69 (↓) *T*_p_: 56.65 ± 0.86 (↓) *T*_c_: 65.48 ± 2.07 (↓) Δ*H*_g_: 0.54 ± 0.04 (↓)	The contents of protein, amylose, and fatty acids were higher in the mutant than in the wild type.	Lee et al. (2017)
*T3612*	11	12.24 ± 0.39 (low amylose)	DP 6–7 ↓, DP 8–13 ↑	–	–	The absence of PDIL1-1 is associated with endoplasmic reticulum stress in the endosperm and underlies floury endosperm formation in the *T3612* mutant.	Han et al. (2012)
*wx* (AeAe/wxwx)	6	0%–0.3% (waxy)	DP ≦ 17 ↓, DP 18–36 ↑, DP ≧ 38 ↑	–	–	The *Waxy* gene encodes GBSS1 and AGPase.	Nishi et al. (2001)

DP, Degree of Polymerization; PT, Pasting Temperature; HPV, hot paste viscosity; PV, peak viscosity; CPV, cool paste viscosity; BD, breakdown; SBV, setback viscosity; CSV, consistent viscosity; GT, gelatinization temperature; To, onset temperature ;Tp, peak temperature; Tc, conclusion temperature; ΔHg, gelatinization enthalpy

### Regulation of starch yield

AGPase catalyzes the first key regulatory step in the starch biosynthetic pathway of higher plants, also referred to as the rate-limiting step. In rice, the *AGPase* gene family contains two small subunit genes (i.e*.*, *OsAGPS1* and *OsAGPS2*) and four large subunit genes (i.e., *OsAGPL1*, *L2*, *L3*, and *L4*). The seed-specific family members were targeted to generate *osagps2* and *osagpl2* mutants, in which lesions of one of two cytosolic isoforms, *OsAGPL2* and *OsAGPS2b*, caused a shrunken endosperm through a remarkable reduction in starch synthesis (Lee et al., 2007). The shrunken rice mutant *w24* (Tang et al., 2016) and the defective grain-filling mutant *gif2* (Wei et al., 2017) exhibited obvious flaws in compound granule formation due to defective *OsAGPL2*. In the absence of *OsAGPL2*, *OsAGPS2b* subunits do not form higher-molecular-mass aggregates (Tang et al., 2016). Also, the *OsAGPL2* subunit is necessary for optimal catalysis and allosteric regulation of the heterotetrameric enzyme (Tuncel et al., 2014). Hence, *OsAGPL2* and *OsAGPS2b* are crucial for normal AGPase activity and starch biosynthesis in the rice seed endosperm (Jeon et al., 2010). The overexpression of *OsAGPases* in rice resulted in improved starch yield due to increased grain weight (Bao et al., 2012), increased AC, and decreased starch breakdown (Lu and Park, 2012). The regulation of *AGPase* is preferred in breeding to increase starch yield. Using TILLING, allelic missense and non-sense mutations were identified in *OsAGPL2*, which altered the allosteric properties of the enzymes and produced shriveled seeds (Tuncel et al., 2014). Interestingly, no natural variations in *AGPases* are being targeted through molecular breeding to improve starch yield.

### Amylose manipulation

It is well known that the *Waxy* gene, encoding granule-bound starch synthase I (GBSSI), controls the synthesis of amylose in the rice endosperm (Hanashiro et al., 2008). GC is negatively correlated with AC and is thus also controlled by the *Wx* locus (Zhang et al., 2018a). Genetic evidence has revealed that *Wx* (*qGC6*) may contribute equally to AC and GC studies (Su et al., 2011). However, until recently, only one quantitative trait locus (QTL), *qGC10* (not a *Wx* allele), had been shown to contribute to GC (Zhang et al., 2020c). Studies have found that functional single-nucleotide polymorphisms (SNPs) in the *Wx* gene are associated with variations in the AC of rice cultivars. To date, at least ten functional variations in the *Wx* locus have been reported to control rice AC within a range of 0%–30%. In non-waxy cultivars, the *Wxlv* and *Wxa* alleles are responsible for the high-AC types (>25%) but lead to differences in starch viscosity (Zhang et al., 2019a). By contrast, *Wx* causes intermediate AC (about 20%), *Wxb* and *Wxmw/la* contribute to low AC (about 14%–15%), and *Wx1-1*, *Wxmp*, *Wxmq*, and *Wxop/hp* contribute to very low AC (8%–12%) (Ando et al., 2010; Zhou et al., 2021a; Zhang et al., 2021b). A 23-bp deletion in the *Wx* locus (*Wx* allele) is responsible for waxy rice (Wanchana et al., 2003). Studies have shown that all these alleles have been selected in response to cultural preferences worldwide (Zhang et al., 2019a; Custodio et al., 2019). Thus, each of the *Wx* alleles has been differentially selected based on regional preferences for rice grain quality improvement to meet consumer needs.

In addition to the direct function of *Wx* in amylose synthesis, dozens of genes are involved in the regulation of the *Wx* gene at the transcriptional and post-transcriptional levels. For instance, *Du1*, *Du3*, *qAC2*, *qSAC3*, *FLO2*, and *LowAC1* have been reported to regulate amylose synthesis directly by manipulating the splicing efficiency of *Wx* mRNA (Zeng et al., 2007; Isshiki et al., 2008; She et al., 2010; Takemoto-Kuno et al., 2015; Igarashi et al., 2021). Some transcription factors, including *OsMADS7*, *OsBP-5*, *OsEBP89*, *REB*, *OsNAC20*, *OsNAC26*, and *NF-YB1-YC12-bHLH144*, have also been found to regulate the expression of *Wx* and other starch synthesis-related genes (*SSRGs*) in rice (Yang et al., 2001; Zhu et al., 2003; Zhang et al., 2018b; Bello et al., 2019; Wang et al., 2020c). In addition, the GBSS-binding protein *OsGBP* was reported to help *GBSSI* locate starch granules in the rice endosperm (Wang et al., 2020a). The CBM48 domain-containing protein *FLO6* was found to regulate starch synthesis in rice by interacting with *GBSS* and other starch synthases. The aforementioned genes only regulate *Wx* expression and are good candidates for rice ECQ improvement.

### Amylopectin modification

In addition to the importance of the amylose to amylopectin ratio for adjusting rice ECQ, the fine structure of amylopectin, especially of the short chains (A and short B chains), also makes a significant contribution to rice ECQ (Li and Gilbert, 2018). Amylopectin synthesis involves multiple isoforms of soluble SSs, SBEs, and starch DBEs (Jeon et al., 2010; Huang et al., 2021a, 2021b, 2021c). SSs are responsible for the extension of the amylopectin branches; they include several isoforms, such as *SSI*, *SSII-1* (*SSIIc*), *SSII-2* (*SSIIb*), *SSII-3* (*SSIIa*), *SSIII-1* (*SSIIIb*), *SSIII-2* (*SSIIIa*), *SSIV-1*, and *SSIV-2* (Jeon et al., 2010; Zhu et al., 2021).

Deficiency in an individual isoform may have a pleiotropic effect on grain development and starch accumulation and will usually lead to low ECQ. Exploring the allelic variation of these genes may be useful for rice grain quality improvement. Although SSI has the most prominent activity of all the SSs, its mutation alone causes no significant change in grain quality. It can be surmised that SSI may therefore participate in the regulation of other SS enzyme activities via multi-enzyme complexes (Crofts et al., 2015). In terms of *SSI* allelic variation, Umemoto et al. (2002) showed that *SSI* variation had a marked effect on starch structural properties (Umemoto et al., 2002). Moreover, rice grains carrying the *SSIj* allele from japonica rice tend to have shorter amylopectin chains and better ECQ than those carrying the *SSIi* allele from indica rice (Luo et al., 2015; Li et al., 2018a).

Among other *SS*-encoding genes, *SSII-1*, *SSII-3* (*ALK*), *SSIII-2*, *SSIV-2*, and *SSIV-3* are preferentially expressed in the endosperm, whereas *SSII-2*, *SSIII-1*, and *SSIV-1* are mainly expressed in the leaves or panicles (Ohdan et al., 2005; Zhang et al., 2021a). *ALK* functions mainly in the synthesis of intermediate amylopectin chains (DP ∼13–24), which can form ordered starch structures and thus contribute to GT properties (Nakamura et al., 2005). The *SSIIa* mutation induced by *N*-methyl-*N*-nitrosourea has been shown to control low GT and may therefore be a good resource for rice ECQ improvement (Miura et al., 2018). Studies have found that at least four *ALK* alleles are associated with GT diversity in rice; *ALKa* and *ALKb* control low GT, whereas *ALKc* and *ALKd* control high GT (Zhang et al., 2020a, 2020b; Chen et al., 2020). Thus, *ALKa* and *ALKb* are good candidates for rice ECQ improvement in breeding programs to optimize cooking temperature. On the other hand, recent studies found that downregulated expression of *SSII-2* led to low expression levels of *ALK* and *Wx*, resulting in low-amylose rice with improved ECQ (Li et al., 2018b; Huang et al., 2021a, 2021b, 2021c). It is noteworthy that mutation of *SSIV-2* or *SSIIIa* had no significant effect on rice starch granule morphology, but their combination resulted in small and spherical starch granules (Toyosawa et al., 2016). Recently, it has been confirmed that the interaction of *AGPlar* and *PUL* contributes to rice ECQ (Xu et al., 2020). The mutation of individual *SS* genes usually leads to undesirable grain quality traits (i.e., *SSIII-1*) and minor changes in ECQ (Huang et al., 2021a; Zhu et al., 2021). The SS enzymes involved in amylopectin synthesis usually act in the form of multi-enzyme complexes. Furthermore, the expression of some *SS* genes may be widely regulated by miRNA functions, especially *miR156s* targeted to *OsSSII-3* (Zhang et al., 2021a). Hence, it is necessary to explore the combined effects of different genes/alleles on rice ECQ improvement.

SBEs, with three isoforms (*SBEI* [SBE1], *SBEIIa* [*SBE4*], and *SBEIIb* [*SBE3*]), function mainly in the production of branches connected by α-1,6-glycosidic bonds. In comparison, DBEs, with four isoforms (*ISA1*, *ISA2*, *ISA3*, and *PUL*), mainly hydrolyze α-1,6-glycosidic bond branches to ensure the orderly synthesis of amylopectin chains (Sawada et al., 2018; Huang et al., 2021a). Studies have found that the downregulation of *SBE1* gene activity has minimal effects on starch synthesis (Satoh et al., 2003), whereas downregulation of the *SBE3* gene leads to a high-amylose phenotype, especially when the expression levels of *SBE3* and *SBE1* are decreased simultaneously (Wang et al., 2017; Sawada et al., 2018). The double mutant deficient in *BEIIb/GBSSI* (*ae/wx*) lacks amylose and has fewer short amylopectin chains (DP ≤ 17) (Nishi et al., 2001). By contrast, the *ss3a/sbe2b rice* double mutant has a very high AC (ca. 45%) and a lower proportion of short amylopectin chains (Takahashi and Fujita, 2017). The gel formed from the *ss3a/sbe2b* mutant had higher GT, rapid retrogradation, and a high hardness level. These mutants may be useful for the confectionery industry because of their potential ability to lend firmness to candies by decreasing hardening times, thereby reducing the manufacturer's cost. CRISPR/Cas9 technology was used for targeted mutagenesis of *BEI* and *BEIIb* in rice, significantly increasing the content of amylose and resistant starch (Sun et al., 2017), which could be used in fiber-fortified foods such as breakfast cereals and cookies because of its nutritional value.

In terms of *DBE*s, the downregulation of *ISA1* was found to cause shriveled kernels (sugary endosperm) with highly branched glucan and phytoglycogen (Utsumi et al., 2011; Du et al., 2018). The reduction of *ISA1* by about 94% in rice endosperm resulted in the accumulation of a water-soluble polyglucan and water-insoluble modified amylopectin instead of the usual amylopectin (Fujita et al., 2003). Compared with the wild type, *sugary1* mutants have different physicochemical properties, such as lower onset GT, lower peak viscosity, and a lesser degree of crystallinity (Wong et al., 2003). *PUL* has more negligible effects on amylopectin biosynthesis (Fujita et al., 2009); however, it can compensate for the role of *ISA* in the construction of amylopectin multiple cluster structures (Kubo et al., 1999). A recent study found that the introduction of higher *SSIIa* activity could primarily complement the sugary phenotype. On the other hand, ISA2 can form heterohexamers with ISA1, which is heat resistant *in vitro*, and the overexpression of *ISA2* leads to shriveled kernels (Utsumi and Nakamura, 2006; Utsumi et al., 2011). *ISA3* was found to facilitate starch metabolism and affect the morphological characteristics of plastids in rice leaves and endosperm (Yun et al., 2011). In addition, the function of *PUL* was shown to partially overlap with that of *ISA1*, and its deficiency has a much smaller effect on grain quality (Fujita et al., 2009). Allelic variation analysis indicated that *PUL* from different glutinous rice accessions resulted in apparent differences in rice ECQ profiles, and the combination of *PUL1* and *SSIV-2c* led to better ECQ (Yan et al., 2011; Xu et al., 2020). The potential industrial applications of rice starch have recently been reviewed (Tiozon et al., 2021).

### Protein

Generally, rice PC can be classified into four fractions according to solubility-linked physical properties: albumins, globulins, prolamins, and glutelins (Kawakatsu and Takaiwa, 2010). Glutelin (including *GluA, GluB, GluC, and GluD)* is the most abundant fraction, and any significant changes in glutelin content will certainly affect rice ECQ (Yang et al., 2019a; 2019b). Great efforts have been made toward dissecting the genetic mechanism underlying rice PC. However, PC is a typical quantitative trait with a complex genetic structure. Furthermore, it is susceptible to environmental factors, especially nitrogen fertilization in the late growth stages, and therefore a limited number of genes/QTLs regulating PC have been identified from various mutants or mapping populations (Pradhan et al., 2019; Yang et al., 2020). For instance, the putative amino acid transporter gene *OsAAP6* was shown to be a positive regulator of rice PC (Peng et al., 2014a). In addition, Wang et al. (2020a, 2020b, 2020c) showed that knockouts of *OsAAP6* and another amino acid transporter gene, *OsAAP10*, could both lead to reduced PC and thus improve rice ECQ (Wang et al., 2020b). The positive PC regulator *OsGluA2*, encoding a glutelin type-A2 precursor, was also isolated, and genome-edited lines exhibited lower PC with improved ECQ (Yang et al., 2019a; 2019b). Allelic variation in the japonica type of *OsGluA2* was also associated with lower PC (Yang et al., 2019a; 2019b). Nitrogenous fertilizers have a significant effect on rice grain PC, and genes related to nitrogen use efficiency play an important role in the transport of amino acids between sources and sinks, thereby affecting protein synthesis. However, little is known about the role of these genes in rice grain PC formation (Zhang et al., 2020c).

### Lipids

Although the lipid synthesis pathway is well understood in other crops (Hernández and Cejudo, 2021), a limited number of genes involved in grain oil synthesis have been identified in rice. Zaplin et al. (2013) identified four putative rice microsomal Δ^12^-fatty acid desaturases (*OsFAD2*). They showed that downregulation of the major *FAD2-1* resulted in increased oleic acid and reduced linoleic and palmitic acids in rice grains (Zaplin et al., 2013). Luo et al. (2021a, 2021b) found that knockdown of *FAD2-1* also influenced starch properties (Luo et al., 2021a). Khan et al. (2020) analyzed the effect of the phospholipase D gene (*OsPLDα1*) on rice grain quality (Khan et al., 2020). They found that the ospldα1 mutants showed reduced AC, lower gelatinization profiles, and thus enhanced rice ECQ (Khan et al., 2020). Recently, Zhou et al. (2021a, 2021b) identified four genes (*PAL6, LIN6, MYR2*, and *ARA6*) encoding enzymes involved in oil metabolism and found that natural variation in these genes contributed to oil composition (Zhou et al., 2021b). Moreover, the genes showed clear differentiation among subpopulations, facilitating marker-based breeding of rice varieties with enhanced oil and grain quality.

### Aroma

Rice fragrance is an important trait that is widely desired among rice producers and consumers. Approximately 300 volatiles have been detected in rice to date, but 2-AP is regarded as the primary aromatic component, and most studies have therefore focused on its genetic regulation. However, the intensity of the aroma conferred by 2-AP is markedly affected by both genetics and the environment (Okpala et al., 2019; Li et al., 2020; Luo et al., 2021b). In fact, the same variety of rice grown under the same conditions may still differ in 2-AP content because of differences in harvest duration and processes. It is well known that *Badh2* (*FGR*) is the dominant gene encoding betaine aldehyde dehydrogenase (BADH2), which inhibits the synthesis of 2-AP, and its non-functional recessive alleles *badh2-E2*, *badh2-E7*, *badh2.1*, *badh2-p-50UTR*, and *badh2-p* are responsible for fragrance in rice (Chen et al., 2008; Kovach et al., 2009; Shi et al., 2013; Bindusree et al., 2017). *Aro3-1* is an aroma QTL that is specific to basmati rice varieties (Amarawathi et al., 2007). Although some scientists have speculated that additional fragrance-causing genes may be associated with fragrance in rice, such new genes are yet to be identified (Fitzgerald et al., 2008). Thus, *Badh2* is currently the only aroma-related target gene in rice breeding programs. Notably, quantitative differences in 2-AP cannot be tightly linked to the marker because of environmental variability, such as high-temperature stress.

## Conventional strategies for rice ECQ improvement

Conventional breeding programs rely mainly on the introgression of existing natural genetic variation into elite backgrounds by tapping rare alleles from diverse genetic resources through marker-assisted breeding (MAB) (Hua et al., 2019; Dheer et al., 2020). In recent years, in addition to improvements in traditional plant breeding through bi-parental crosses, three-line and two-line hybrid breeding to increase heterosis and double haploid technology to fix alleles in early generations have been deployed to improve grain yield and quality (Breseghello and Coelho, 2013; Ahmar et al., 2020). All these various breeding approaches require effective phenotypic screening for rice grain quality traits to advance breeding selections, and these screens are time consuming, costly, and low throughput in nature. By contrast, breeders would greatly benefit from molecular markers linked to specific target traits. Traditional molecular genetic studies have contributed to our understanding of molecular regulation mechanisms, and germplasm resources with trait-enhancing alleles associated with rice ECQ have been widely identified. Competitive allele-specific PCR (KASP) markers, derived from the fluorescence-based PCR detection of SNPs and small insertions and deletions (InDels), have been widely used in various breeding programs (Yang et al., 2019a; 2019b). Gene-specific molecular markers (functional markers) are stable, cost-effective, and easy to use for MAB, and their extensive capabilities have made them a potent tool for rice breeding selection (Phing Lau et al., 2016; Ahmar et al., 2020; Salgotra and Stewart, 2020).

There have been a number of successful cases of rice ECQ improvement by MAB over the years. The ECQ of cooked rice depends largely on AC, and many genes that reduce the AC of rice grain have been identified and used to improve cooked rice ECQ in order to meet market demands. In Japan, the japonica rice variety Yumepirika, which harbors the *Wx1-1* allele and exhibits sticky eating quality, was developed and evaluated as a premium variety for the Japanese market (Fujino et al., 2019). Moreover, some desirable QTLs that contribute to low AC have been identified and utilized for ECQ improvements through MAB in Japan (Fujino et al., 2019). In China, molecular marker-assisted selection (MAS) of the *Wxb* allele has been used to improve grain quality for a long time, and most indica rice now carries the *Wxb* allele (Yi et al., 2009; Shao et al., 2020), whereas the *Wxa* allele has been targeted in South Asia (Anacleto et al., 2019). The *Wxmp* allele that controls low AC has been successfully used for low AC breeding in China by MAS, and a series of japonica varieties (i.e., Nanjing 46, Nanjing 5055, and Nanjing 9108) with low AC (10%–15%) have been bred (Wang et al., 2017, 2021). The *Wxmw/la* allele has also proven to be a valuable candidate for rice ECQ improvement because of its contribution to low AC (∼14%) (Zhou et al., 2021a; Zhang et al., 2021c). In addition to direct introgression of the *Wx* allele, some genes that regulate the *Wx* locus have also been used in MAB. The *qSAC3* locus from indica rice has been valuable for fine-tuning rice AC, improving the appearance quality of soft rice with low AC (Zhang et al., 2019b). Genes/QTLs directly associated with the regulation of amylose synthesis are potential candidates for rice ECQ improvement by MAS.

To manipulate rice amylopectin, genes such as *SSI* and *ALK* have proven to be good targets for rice ECQ improvement by MAS. The *SSIj* allele from japonica rice has been reported to lead to shorter amylopectin chains when introduced into indica rice, resulting in better ECQ (Luo et al., 2015; Li et al., 2018b). Allelic variation in *ALK* is responsible for GT variation among varieties, and rice grains with low GT usually have better ECQ, particularly after the cooked rice has cooled (Zhang et al., 2020c). Our previous studies have shown that the introduction of *ALKa* and *ALKb* to japonica rice leads to better ECQ relative to rice carrying the *ALKc* allele, contributing to high GT (Chen et al., 2020). Other SSRGs contribute to amylopectin synthesis; however, they have shown only minor effects on rice ECQ (Tian et al., 2009). The introduction of multiple allele combinations by MAS is a possible approach for producing large changes in ECQ. For example, through rational design, the introduction of *SSRG* alleles (*Wx*, *ALK*, *AGPL1*, *AGPS2a*, *SSI*, *SSIII-2*, *SSIV-2*, *SBE3*, *PUL*, and *ISA*) from high-quality parents to low-quality parents produced superior-quality rice varieties (Zeng et al., 2017).

For the manipulation of rice PC, aroma, and lipids, target genes such as *OsAAP6*, *OsGluA2*, *badh2*, *PAL6*, *LIN6*, *MYR2*, and *ARA6* are potential candidates for rice ECQ improvement by MAS. Reducing rice PC has become another important target for the breeding of rice varieties with good ECQ. For example, introgression of the QTL *qPC-1* (indica type) into a japonica background leads to a decrease in PC, thereby enhancing ECQ (Yang et al., 2015). Although numerous QTLs for GPC variation have been detected in rice in the past decade, few QTLs have been cloned, with the exception of *qPC1/OsAAP6* and *OsGluA2* (Peng et al., 2014a; Yang et al., 2019a, 2019b). The expression level of *OsAPP6* is associated with PC variation only in indica accessions. Thus, the low expression allele of *OsAPP6* may be a good target gene for generating low-PC grain in indica rice by MAS. In contrast to *OsAAP6*, the two haplotypes of *OsGluA2*, *OsGluA2LET* and *OsGluA2HET*, are found mainly in japonica and indica cultivars, respectively. The *OsGluA2LET* allele exhibits lower transcription and is thus a promising target gene for low-PC rice breeding through MAS (Yang et al., 2019a; 2019b). Gene-specific molecular markers such as *NKSbad2*, *FMbadh2-E7*, *BADEX7-5*, *Aro7*, and *KASP* markers (nine SNPs) that are highly associated with elevated 2-AP content have been used for aroma improvement by MAS (Golestan Hashemi et al., 2015; Addison et al., 2020). In terms of lipid manipulation, although the four genes *PAL6*, *LIN6*, *MYR2*, and *ARA6* have been found to contribute to lipid content variation among rice accessions, there is no evidence for their use in MAB programs.

## Modern molecular breeding strategies for rice ECQ improvement

The availability of rare beneficial alleles in nature limits the effective deployment of target traits in conventional crop breeding. To overcome these limitations, mutation breeding has been developed to introduce non-naturally occurring alleles generated by random mutagenesis using physical, chemical, and biological means. The initial mutagenesis must be followed by screening large populations to identify mutants with desirable grain quality properties. An *N*-methyl-*N*-nitrosourea-based mutagenesis method was used to identify high-amylose and highly resistant starch mutants by combining indica *SSIIa* and indica *GBSS* genes with japonica *SBEIIb* alleles deficient in *sbeIIb* activity (Itoh et al., 2017). In addition, a *floury endosperm 8* mutant deficient in UDP-glucose pyrophosphorylase with impaired starch structure was isolated (Long et al., 2017). γ-ray-induced mutations resulted in the identification of a GM645 high-amylose line (Kong et al., 2015) and an aromatic line with increased 2-AP content (Sansenya and Wechakorn, 2021).

Genetic engineering and plant transformation technology have played a pivotal role in crop improvement by enabling the introduction of beneficial foreign gene(s) or the silencing of endogenous gene(s) in crop plants (Kumar et al., 2020). In rice ECQ improvement, targeted gene silencing is an effective tool to manipulate biosynthetic pathways in a constitutive or tissue-specific manner to obtain the desired phenotype. For example, the *Wx*, *SSI*, *SSII-2*, *ALK*, *FAD2-1*, and *Badh2* genes were suppressed by RNA interference, and the resulting transgenic rice showed improved ECQ traits compared with the wild type (Li et al., 2018a; Zhao et al., 2019; Chen et al., 2020; Adegoke et al., 2021). However, widespread adoption of transgenic crops carrying foreign genes faces roadblocks due to regulatory concerns about potential toxicity and allergenicity to humans, adverse effects on non-target organisms, evolution of resistant weeds and insects, and so forth. Thus, recent innovations in next-generation clustered regularly interspaced short palindromic repeat/CRISPR-associated (CRISPR/Cas) systems, such as prime editing and base editing, have promoted the idea that genome editing is revamped for crop improvement (Kumar et al., 2020). Because genome-edited crop plants are free from foreign genes, they are expected to gain greater consumer acceptance than transgenic crops and to obtain faster regulatory approvals.

Gene-editing technology is now being widely applied and has been used to directly generate new rice plants with improved ECQ. For the manipulation of rice starch, a series of novel *Wx* alleles have been created by editing the promoter and coding region of rice *Wx* using the CRISPR/Cas system, and the new *Wx* alleles contribute to moderate AC, which is a potential target for rice ECQ improvement (Huang et al., 2020a, 2020b, 2021b; Gao et al., 2020). To manipulate PC, two amino acid transporter genes, *OsAAP6* and *OsAAP1*, were knocked out through gene editing, and grains from the mutated plants had lower amino acid content and PC and therefore improved ECQ (Wang et al., 2020b). For aroma improvement, the fragrance gene *Badh2* has been edited by CRISPR/Cas9 mutagenesis in different rice cultivars (including three-line hybrid rice), and a series of non-functional *badh2* alleles have been generated (GaoNeng et al., 2017; Ashokkumar et al., 2020; Hui et al., 2021; Tang et al., 2021). In addition, *fatty acid desaturase 2* (*FAD2-1*) was also knocked out by gene editing. The mutant rice showed high unsaturated fatty acid composition, which may lead to ECQ improvement (Abe et al., 2018). A limited number of favorable genes/alleles (i.e., *Du1*, *Du3*, *qAC2*, and *LowAC1*) are directly responsible for rice ECQ, and these individual genes are good targets for rice ECQ improvement using a gene-editing system.

In fact, most improvements in ECQ traits reported to date have been obtained by manipulating only a single gene. It should be noted that ECQ is a complex trait that reflects softness, aroma, extra elongation, and other characteristics. Thus, the manipulation of individual genes may be insufficient for ECQ improvement. Our previous studies have shown that the coordinated expression of *SSII* and *Wx* is a good approach for rice ECQ improvement (Huang et al., 2021c). Moreover, to improve crop characteristics, many genes that regulate essential traits must have a high translation rate rather than functional loss or reduction (Reis et al., 2020). Recently, the modification of *cis-regulatory elements* has been shown to be a practical approach for fine-tuning the expression levels of target genes and is therefore a promising strategy for rice ECQ improvement (Ding et al., 2021).

## Fostering genomics-aided information to improve ECQ, texture, and palatability

The main means of ensuring wider rice acceptability is the development of high-yielding varieties that meet the ECQ and textural preferences expressed by consumers. Although high rice amylose levels (≥25% AC) contribute to the hardness of cooked rice, fully characterizing rice textural properties requires measurements beyond AC. Mining the large germplasm resources of indica lines in the IRRI breeding program revealed that high-amylose lines may also exhibit relatively soft GC. This finding implies that some high-amylose varieties remain soft upon cooling (Anacleto et al., 2015). Milled rice endosperm is typically composed of 90% starch, but many varieties differ in the composition of amylose and amylopectin. Hence, starch composition is expected to contribute to differences in texture, making the link between starch structure and ECQ/texture a pivotal one. Modeling tools, such as random forest and artificial neural networks, based on pasting properties (pasting temperature, peak viscosity, and FV) and amylose and amylopectin composition were used to identify 12 distinct ideotypes of rice ECQ properties. Variation in short-chain amylopectin regions (SCAP3, SCAP2, and SCAP1) and amylose regions (AM2 and AM1) was found to distinguish among ECQ classes in a rice diversity panel (Buenafe et al., 2021).

In recent years, rapid technological advances in genotyping and phenotyping platforms have facilitated multi-trait association studies via genome-wide association studies (GWAS). This technique can accurately document the genetic make-up of rice ECQ based on marker–trait associations, including traits such as starch structural composition, textural attributes, and rapid viscosity analysis parameters (Butardo et al., 2017; Misra et al., 2018; Buenafe et al., 2021; Hori et al., 2021; Wei et al., 2021). Interestingly, amylose showed a strong negative correlation with adhesiveness, and a fine-mapped region of chromosome 6 was strongly associated with both AC and adhesiveness (including LOC_Os06g04169, LOC_Os06g04200, LOC_Os06g04530, and the intergenic region covering LOC_Os06g38564–LOC_Os06g38580). AC showed weaker correlations or no correlation with the remaining textural attributes, such as hardness, cohesiveness, and springiness, but multi-locus GWAS identified at least 97 marker–trait associations for these textural properties that were validated as minor QTL contributing factors by two independent multi-locus GWAS methods (Misra et al., 2018).

Rapid viscosity analysis is a reliable technique that generates amylographs with reproducible phenotypic data that are useful for distinguishing soft rice within high-amylose classes. The measured parameter is FV (i.e., the viscosity at the end of the run, corresponding to the amylograph of cool paste viscosity), which reflects cooking quality properties. FV has been shown to accurately discriminate waxy from non-waxy samples and retrograded from non-retrograded samples (Buenafe et al., 2021). FV reflects the influence of starch gelatinization and the viscosity properties of cooked rice. The diversity in FV among 625 re-sequenced genomes from the 3000 Rice Genomes Project was recently inspected, and its underlying genetic basis was revealed in a GWAS using 3 401 089 high-density SNP markers. The hotspot SNPs influence FV in indica rice, and four clusters of genes near *GBSSI* on chromosome 6 are estimated to be in eight linkage disequilibrium blocks that contribute to differences in the FV/amylose ratio. *GBSSI* and neighboring unknown genes were found to have the greatest influence on FV; the most significant SNP was located at the splice junction of intron 1, and five more significant SNPs were found in the promoter region and exons 9 and 10 of *GBSSI* (Anacleto et al., 2019). These target SNPs were strongly selected during domestication to alter cooking quality in japonica and indica subtypes. The identification of other functionally relevant SNPs in regions of chromosomes 1, 2, 3, and 11 confirms the presence of other genes that make a minor contribution to the variation of cooked rice quality in indica versus japonica subtypes.

The high-AC *Wx* alleles typically confer poor palatability but are beneficial for preventing type II diabetes, obesity, and colorectal cancer, as AC is positively correlated with resistant starch (Jukanti et al., 2020). To overcome this limitation, Anacleto et al. (2019) combined a GWAS with a transcriptome-wide association study (TWAS). They identified the *Wxa* (G allele) at the first exon/intron boundary of *GBSSI*, an alternative splice site associated with intermediate to high GI variation. An additional SNP (C˃T) at exon 10 was found to influence FV, independent of amylose content and GI. The combination of these two SNPs produced the GC haplotype, which explained the phenotype of intermediate GI with soft texture (intermediate FV). By contrast, the GT haplotype was associated with intermediate GI and hard texture (high FV). The TC haplotype confers high GI and a softer texture in rice from Thailand, Myanmar, and Laos. Notably, cooking quality classes, as explained by these two SNP haplotypes, neatly classified rice lines according to cultural preferences. Germplasm from Southeast Asian countries possessed the GC combination with a soft rice texture, whereas germplasm across the Pacific from Mainland Asia and South American countries predominantly possessed the GT haplotype. It is noteworthy that clades with GC or GT haplotypes were present in germplasm from India and Bangladesh. Thus, selecting lines based on AC alone may lead to difficulty in predicting the right texture, owing to allelic variance introduced in these two SNPs (Anacleto et al., 2019). These haplotypes and target genes have been validated using TWAS and gene regulatory network approaches. The target genes are recommended for functional validation, as they are potentially useful for precision breeding. The regulation of the *GBSSI* complex involves transcriptional and post-transcriptional modification. TWAS and methylome sequencing data revealed *cis*-acting functionally relevant genetic variants with differential methylation patterns in the *GI6.1* hotspot region, suggesting a role for DNA methylation in the regulation of *GBSSI* expression (Anacleto et al., 2019). With revolutionary advances in DNA/RNA sequencing methods, genome-wide SNP arrays, genotyping-by-sequencing approaches, and high-throughput SNP markers (i.e., KASP) have become cost-effective approaches for genomic selection and can now be integrated into breeding schemes.

## Perspective

**Figure 3:**
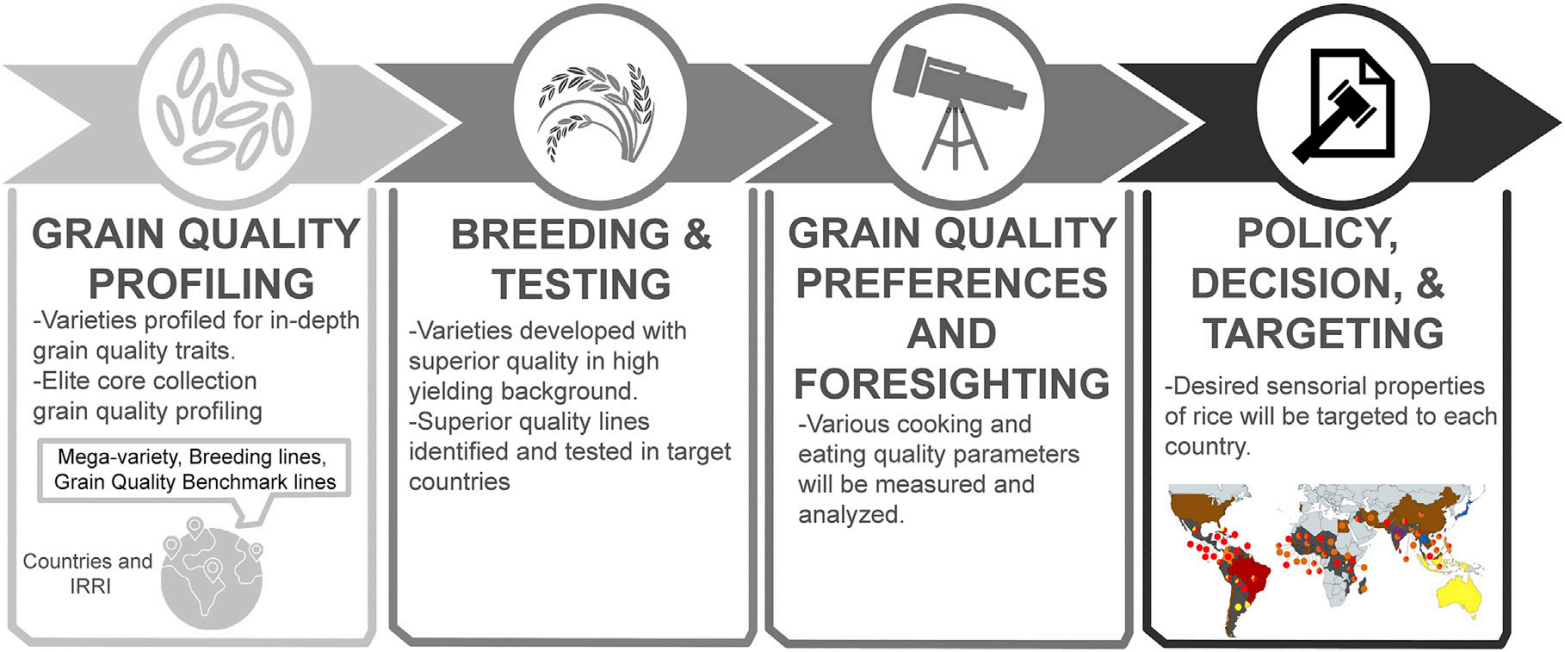
Premium quality monitoring for the rice industry.

Conventional breeding strategies (including MAB) for crop improvement have been used for many years. However, their efficiency is limited because of genetic drag, genetic erosion, hybridization bottlenecks, and laborious selection processes. It can take several years to develop a crop variety with desired traits, making it a complicated and time-consuming endeavor. With recent advances in molecular biology and the discovery of new genes/QTLs related to rice grain qualities, biotechnologists can now manipulate an organism's genome in a precise way with the aid of CRISPR and its associated Cas proteins. Next-generation CRISPR/Cas systems, such as prime editing, base editing, and de novo domestication, have revolutionized genome editing for crop improvement. New approaches are now possible, such as genomic-assisted breeding strategies that not only capture major QTL factors but also enrich minor allele factors through population-based improvement to augment ECQ traits (i.e., texture, elongation properties, and aroma). The design of elite × elite core collections with high genomic estimated breeding values for high yield potential with ECQ and superior-quality donor lines, together with efficient genomic selection breeding tools, will ensure the development of high-yielding varieties with appropriate textures. By employing new precision breeding approaches, future crop breeding can meet the consumer demands of a rapidly increasing population in the coming decades. In addition, modeling techniques can be deployed in combination with genomic selection through advanced population breeding to fine-tune trade-offs between nutritional attributes, such as lower glycemic index and increased protein content, and ECQ traits. This approach can lead to the identification of optimum alleles that fulfill the demands of rice value chain stakeholders and produce premium quality varieties. It will enable future breeding programs to efficiently mine the huge genetic diversity of rice for novel genes that enhance rice food quality. The integration of multi-tiered fingerprinting data (grain quality, sensory, and value chain inferences) is essential for improving the efficiency of breeding operations. It makes full use of genetics and genomics to ensure that advanced breeding materials match the grain quality segments of mega varieties with superior grain quality and high yield potential. To disseminate breeding material to target countries, we must fingerprint the grain quality and ECQ traits of advanced breeding material and deploy modeling tools to identify the classes that match the mega varieties preferred in the target countries (Figure 3). Such genomics-driven information, combined with state-of-the-art grain quality and sensory-based phenotyping methods, aids in the dissemination of genetic materials to ensure better acceptance by stakeholders in the rice value chain.

## Funding

N.S. acknowledges funding support from the RICE CGIAR Research Program and AGGRI project (grant no. OPP1194925) from the 10.13039/100000865Bill and Melinda Gates Foundation. Q.L. and N.S. appreciate the funding support of the 10.13039/501100001809National Science Foundation of China (grant nos. 32161143004 and 31825019).

## Author contributions

Conceptualization, N.S; writing – original draft, N.S., C.Z., R.N.T., and Q.L.; writing – review & editing, N.S. and Q.L.; visualization, R.N.T.; supervision, N.S. and Q.L.
